# The Book World of Medicine and Science

**Published:** 1896-12-05

**Authors:** 


### The Book World of Medicine and Science.

Premature Burial, and how it may be Prevented. By William Tebb, F.R.G.S., and Col. Edward Perry Vollum, M.D. (London: Swan Sonnenschein and Co. 1896. Price 5s.)

The object of this book is to maintain the thesis that decomposition is the only reliable test of death, that premature burial is far more common than is imagined, and that the only way of preventing such an occurrence is to delay burial until the commencement of decomposition. The corollary of this is obvious, viz., that "waiting mortuaries" should be established, not dead houses, but warm, light, well ventilated places, in which the bodies of those who are supposed to be dead should be deposited to await events, and be kept until decomposition sets in. It is easy to agree with this conclusion without attributing as much importance as the authors do to many of the statements they have collected. In saying this, however, we would not wish in any way to disparage the interest of the book. It is, undoubtedly, sufficiently horrible, and considerable evidence, such as it is, is brought forward as to premature burial being of much greater frequency than many people have supposed to be the case. The stories as to the narrow escapes that some people have had are, perhaps, the most interesting part of the book, and if not always very authentic, at the least are *ben trovato*. Some of the best, if one may so say, have reference to soldiers in India during times of cholera; but the tales have been collected from many sources, the instances of narrow escape including, among others, that of Madame Blavatsky. A sketch is given of some of the Continental and American systems of verification of death, but the conclusion is arrived at that in practice they are not reliable, "the only safe and infallible test of dissolution being the manifestation of putrefaction in the abdomen." It is therefore urged that "the only safe and effective method of reform is the establishment of appropriately designed waiting mortuaries, such as are provided at Munich, Weimar, Stuttgart, and other German cities, with qualified attendants and appliances for resuscitation," where doubtful cases may be deposited until the fact of death is unequivocally established. The book is nicely got up, and is of a popular readable character. It is not a medical book, although it contains many extracts from medical papers.

